# C6orf15 promotes liver metastasis via WNT/β-catenin signalling in colorectal cancer

**DOI:** 10.1186/s12935-024-03324-2

**Published:** 2024-04-23

**Authors:** Jiankang Yu, Jian Sun, Jingtong Tang, Jiayu Xu, Guanru Qian, Jianping Zhou

**Affiliations:** 1 Department of Gastrointestinal Surgery & Hernia and Abdominal Wall Surgery, The First Hospital, China Medical University, Shenyang, 110001 China; 2 Shenyang Medical Nutrition Clinical Medical Research Center, Shenyang, China

**Keywords:** Colorectal cancer, Epithelial–mesenchymal transition, Liver metastasis, WNT/β-catenin signalling, Fatty acid metabolism, C6orf15

## Abstract

**Background:**

Colon cancer ranks third among global tumours and second in cancer-related mortality, prompting an urgent need to explore new therapeutic targets. C6orf15 is a novel gene that has been reported only in Sjogren’s syndrome and systemic lupus erythematosus patients. We found a close correlation between increased C6orf15 expression and the occurrence of colon cancer. The aim of this study was to explore the potential of C6orf15 as a therapeutic target for colorectal cancer.

**Method:**

RNA-seq differential expression analysis of the TCGA database was performed using the R package ‘limma.’ The correlation between target genes and survival as well as tumour analysis was analysed using GEPIA. Western blot and PCR were used to assess C6orf15 expression in colorectal cancer tissue samples. Immunofluorescence and immunohistochemistry were used to assess C6orf15 subcellular localization and tissue expression. The role of C6orf15 in liver metastasis progression was investigated via a mouse spleen infection liver metastasis model. The association of C6orf15 with signalling pathways was assessed using the GSEA-Hallmark database. Immunohistochemistry (IHC), qPCR and western blotting were performed to assess the expression of related mRNAs or proteins. Biological characteristics were evaluated through cell migration assays, MTT assays, and Seahorse XF96 analysis to monitor fatty acid metabolism.

**Results:**

C6orf15 was significantly associated with liver metastasis and survival in CRC patients as determined by the bioinformatic analysis and further verified by immunohistochemistry (IHC), qPCR and western blot results. The upregulation of C6orf15 expression in CRC cells can promote the nuclear translocation of β-catenin and cause an increase in downstream transcription. This leads to changes in the epithelial–mesenchymal transition (EMT) and alterations in fatty acid metabolism, which together promote liver metastasis of CRC.

**Conclusion:**

Our study identified C6orf15 as a marker of liver metastasis in CRC. C6orf15 can activate the WNT/β-catenin signalling pathway to promote EMT and fatty acid metabolism in CRC.

**Supplementary Information:**

The online version contains supplementary material available at 10.1186/s12935-024-03324-2.

## Introduction

Colorectal cancer (CRC) is the third most common cancer worldwide and the second leading cause of cancer-related deaths. Despite improvements in survival rates, metastatic CRC remains a lethal disease, with a 5-year survival rate of approximately 14% [1]. The liver is the most common site for distant metastasis in patients with CRC, with approximately 50% of patients eventually experiencing liver metastases during the course of the disease [2, 3]. Hence, exploring novel biomarkers and therapeutic targets for liver metastasis in CRC has been a subject of considerable interest.

Chromosome 6 open reading frame 15 (C6orf15), also known as the ape taste bud-specific gene (STG), is located at chromosome 6p21 in humans [4].C6orf15 is a protein-coding gene and is a secreted protein, with a subcellular localization mainly in the extracellular matrix; it is predicted to participate in extracellular matrix composition and potentially facilitate diverse functions, including collagen-V-binding activity, fibronectin-binding activity, and glycosaminoglycan-binding activity. The exact function of C6orf15 has not been elucidated, and there is little related information. Previous reports have shown that this gene is associated with skin diseases and systemic lupus erythematosus [5, 6]. For malignant tumours, C6orf15 may be a novel biomarker for hepatocellular carcinoma [7], but the role of this gene in other types of tumours deserves further exploration. In this study, we conducted differential expression analysis using the TCGA database and included 312 patients with colorectal cancer without distant metastasis and 58 patients with distant metastasis. We identified C6orf15 as the most significantly differentially expressed gene (|logFC|> 1, p value < 0.05). The Hallmark database was used for enrichment analysis of signalling pathways, identifying C6orf15 as significantly associated with the EMT phenotype of CRC. Therefore, we investigated how high C6orf15 expression affects the distant metastasis of CRC. A series of meaningful findings were obtained, laying a solid foundation for us to consider C6orf15 as a potential target for the treatment and prevention of the malignant progression and adverse prognosis of CRC.

The WNT/β-catenin signalling pathway is a prominent pathway in disease research. Dysregulation of the WNT pathway is intricately linked to almost all stages of tumorigenesis in a variety of cancers [8], especially in CRC, where it promotes in vivo colorectal cancer cell invasion and migration, subcutaneous tumour growth, angiogenesis, and liver metastasis [9]. In our experiments, we observed that the upregulation of C6orf15 expression promoted the nuclear translocation of β-catenin, leading to downstream TCF/LEF1 transcription and facilitating the epithelial–mesenchymal transition (EMT) in CRC. Surprisingly, the upregulation of C6orf15 expression induced changes in fatty acid metabolism. Previous studies have shown that fatty acid metabolism is closely associated with the metastatic process of CRC. Additionally, following the upregulation of C6orf15 expression, the expression of the key enzyme CPT1A in the fatty acid metabolism pathway increased; notably, our assessments of fatty acid oxidation metabolism confirmed this outcome. Therefore, we speculate that the upregulation of C6orf15 expression may also affect CRC metastasis by affecting fatty acid metabolism.

## Materials and methods

### Human specimens

The RNA sequencing dataset of CRC patients was downloaded from TCGA. Patients with missing survival and metastasis data were excluded. A total of 370 CRC patients, including 312 without distant metastasis and 58 with distant metastasis, were included in the study.

We obtained 150 pairs of fresh CRC tissues and their corresponding adjacent nontumor colorectal tissues from patients (87 males and 63 females; median age, 63 years; age range, 29 to 81 years). The samples were collected at the Department of Gastrointestinal Surgery, the First Affiliated Hospital of China Medical University. All specimens were pathologically diagnosed as CRC and classified using the guidelines of UICC article 8th. After excluding patients with incomplete clinical data, the patients were divided into high expression and low expression groups according to the median expression of C6orf15, and the correlation between its expression and clinicopathological characteristics such as age, gender, tumor size, differentiation, lymph node metastasis, distant metastasis were analyzed. All studies were conducted in accordance with the Declaration of Helsinki and approved by the Ethics Committee of the First Hospital of China Medical University. In addition, informed consent was obtained from each patient.

### Cell lines

Two human-derived CRC cell lines (HCT116 and RKO) and one C57B6 murine-derived CRC cell line (MC38) were obtained from the cell bank of the Chinese Academy of Sciences (Shanghai, China). HCT116 and MC38 cells were cultured in DMEM containing 10% foetal bovine serum (FBS; Hy Clone, Logan, UT, USA) and 100 U/ml penicillin‒streptomycin (SH30243.01, Hy Clone, Logan, Utah, USA) in a humidified incubator with 2% CO_2_ at 37 °C. RKO cells were cultured in RPMI-1640 (11875093, Gibco, Waltham, MA, USA) supplemented with 10% FBS. All cell lines used in this study were validated by short tandem repeat (STR) genotyping.

### Differential expression analysis and gene set enrichment

RNA-seq differential expression analysis of the TCGA database was performed using the R package “limma”. Differentially expressed genes between tumour samples with and without distant metastases were defined as upregulated or downregulated genes with a fold change > 1 and a false discovery rate (FDR) of P < 0.05. The gene sets were identified by gene set enrichment analysis (GSEA) of the Hallmark gene set of “MSigDB” (Molecular Signatures Database, version 6.0). In addition, the enrichment score was calculated by determining the expression of the gene set. We considered a gene set to be “enriched” when the majority of its expression was either elevated or reduced. GEPIA was used for the correlation analysis of target genes and survival.

### RNA isolation and quantification by real-time fluorescence quantitative PCR

Total RNA from CRC cell lines and 48 colorectal tissue samples was extracted with TRIzol reagent (Takara Bio, Otsu, Japan) according to the manufacturer’s instructions and then stored in liquid nitrogen. A NanoDrop ND-1000 instrument (NanoDrop, USA) was used to measure the total mRNA concentration, and an RT‒PCR quantification kit (Shanghai, China) was used for reverse transcription. qRT‒PCR was performed using a SYBR real-time PCR kit (Takara) with Quant Studio 6 Flex according to the manufacturer’s instructions, with GAPDH serving as an internal control. The thermal cycling conditions were 95 °C for 3 min, followed by 45 cycles of 95 °C for 12 s and 62 °C for 45 s. The following primers were used: C6orf15 forward primer TGCTCCTGGTCTGTCTTCATCTCC and reverse primer GGCTGCGGATGTTCAGAGTTAGAG. The GAPDH internal primers were purchased from Gemma Genetics (Shanghai, China). Each experiment was performed three times in duplicate.

### Protein isolation and western blotting

Total protein was extracted from three infected CRC cell lines (HCT116, RKO, and MC38) using RIPA lysis buffer supplemented with 1% PMSF. Total protein was separated using a 10% sodium dodecyl sulfate‒polyacrylamide gel and then transferred to a PVDF membrane. The membranes were blocked with 5% skim milk for 2 h and then incubated with rabbit anti-C6orf15 (Proteintech, USA; 1:1000 dilution), rabbit anti-β-catenin (Proteintech, USA; 1:1000 dilution), rabbit anti-ZEB1 (Abmart, Shanghai; 1:1000 dilution), rabbit anti-E-cadherin (Abmart, Shanghai; 1:1000 dilution), rabbit anti-N-cadherin (Abmart, Shanghai; 1:1000 dilution), rabbit anti-Vimentin (Proteintech, USA; 1:1000 dilution), rabbit anti-ZO-1 (Abmart, Shanghai; 1:1000 dilution), mouse anti-GAPDH (Abmart, Shanghai; 1:3000 dilution), rabbit anti-CPT1A (Proteintech, USA; 1:1000 dilution), and rabbit anti-LaminB1 (Proteintech, USA; 1:1000 dilution). The membranes were then incubated with secondary antibodies for 2 h. We assessed the protein blotting results using an enhanced chemiluminescence (ECL) assay kit (Thermo Fisher Scientific, Rockford, IL, USA), and each experiment was performed three times.

### Immunohistochemical (IHC) staining

Consecutive 4-μm paraffin-embedded sections of CRC tissue were prepared, deparaffinized at 65 °C for 2 h, and then washed with PBS. Antigen retrieval was performed under high-temperature, high-pressure conditions for 3 min. Subsequently, the sections were incubated with hydrogen peroxide (3%) or 10% normal goat serum for 15 or 20 min, respectively. The sections were then incubated overnight at 4 °C with C6orf15 antibodies (CST, USA; 1:200), Vimentin antibodies (Proteintech, USA; 1:200), and ZEB1 (Proteintech, USA; 1:200). The sections were incubated with peroxidase-conjugated streptavidin–biotin complex for 15 min, followed by the addition of 3,3'-diaminobenzidine (DAB) for colour development. Visualization was performed at 20 × magnification. Immunohistochemistry (IHC) scores were calculated as the product of the staining area (< 5%, 0 points; 5–25%, 1 point; 26–50%, 2 points; 51–75%, 3 points; and 76–100%, 4 points and staining depth (no colour, 0; light yellow, 1; tan-yellow, 2; and brown‒yellow, 3). The total score for each section was further classified into four intensity grades: 0, negative (−); 1–4, weakly positive ( +); 5–8, positive (+ +); and 9–12, strongly positive (+ + +).

### Establishment and transfection of stable cell lines

The C6orf15-overexpressing lentivirus, C6orf15-RNAi-Easy lentivirus and their corresponding negative controls (NCs) were synthesized by GENECHEM (Shanghai, China), and for transfections, Lipofectamine 3000 (Invitrogen, Carlsbad, CA, USA) was used according to the manufacturer's instructions. To establish stable negative control and C6orf15 knockdown cells (HCT116-C6orf15-NC/KD, RKO-C6orf15-NC/KD), puromycin (P8230, Solarbio, Shanghai, China) was used to screen for cells in which C6orf15 was successfully knocked down. Similarly, negative control and C6orf15-overexpressing cells (HCT116-C6orf15-NC/OE, RKO-C6orf15-NC/OE, and MC38-C6orf15-NC/OE) were generated via the same method. The transfection efficiency was also assessed.

### Cell migration assay

As described previously, stable C6orf15 overexpression, C6orf15-knockdown and negative control HCT116 and RKO cell lines were constructed. A total of 8 × 10^4 stable HCT116 cells and 5 × 10^5 stable RKO cells in 300 µl of serum-free medium was added to the upper chamber of a Transwell, and 600 μl of medium containing 10% foetal bovine serum was added to the lower chamber. CRC cells in the bottom membrane were fixed using cold methanol after 24 h and then stained with 0.1% crystal violet (Sigma) for 30 min. Photographs were taken using an EVOS automated imager, and the number of colonies in five random areas in each chamber were counted at × 20 magnification.

### MTT assay to assess the viability of CRC cells

Cells were cultured in 96-well plates (5 × 104 cells/well). Twenty-four hours after plating, the medium was replaced with fresh medium containing 15 μL of MTT. After four hours of incubation at 37 °C, the medium was discarded, and DMSO (150 μL) was added to each well. Then, the absorbance was measured at 490 nm. The results are presented as the percent inhibition compared to that of the untreated control.

### Immunofluorescence

Tissue sections of liver metastases and primary foci of colon cancer from the same patient were placed in xylene, anhydrous ethanol, 95%, 85%, and 75% alcohol in series and in water for deparaffinization and then placed in citric acid antigen repair solution. Next, the sections were incubated in 4% BSA for 30 min before they were incubated with a primary antibody [rabbit anti-C6orf15 (Proteintech, USA; 1:100 dilution)] at 4 °C overnight. The sections were then incubated with a fluorescent secondary antibody (Proteintech, USA). Nuclei were visualized using DAPI (Sigma, St. Louis, MO, USA), and the sections were analysed using an EVOS fully automated imager (Invitrogen, USA).

### Establishment of a mouse model

Female 6- to 8-week-old C57B6 mice were purchased from Changsheng (Benxi, Liaoning, China). Mouse-related studies were performed in specific pathogen-free facilities, and all procedures were approved by the Institutional Animal Care and Use Committee of China Medical University (Shenyang, China), which complies with the National Institutes of Health regulations for the care and use of laboratory animals.

To establish a CRC liver metastasis mouse model, 1 × 10^6^ MC38 cells overexpressing C6orf15 (MC38-C6orf15-OE) or control cells (MC38-C6orf15-NC) were injected into the spleens of C57B6 mice. At 20 days after injection, the mice were euthanized by cervical dislocation, and liver specimens were collected and photographed to record the size and number of metastatic lesions.

### Seahorse extracellular flux analysis

Fatty acid oxidation (FAO) content was determined using a hippocampal XF96xe extracellular flux analyser (Agilent Technologies, North Billerica, MA, USA). The constructed C6orf15-overexpressing RKO CRC cells were seeded into XF96 plates containing DMEM supplemented with 10% FBS at a density of 30,000 cells/well and incubated for 2 days. Then, the cells were collected for a fatty acid oxidation (FAO) assay. Etomoxir, an inhibitor of carnitine ester acyltransferase-1 (CPT-1) and thus FAO, was added to the cells prior to the assay with other drugs in accordance with the instructions of the long-chain fatty acid oxidative metabolism pressure kit and preadded to the dosing wells. The specific dosing concentrations used were 4 µmol/L Eto, 1.5 µmol/L oligomycin, 1.0 µmol/L FCCP, and 0.5 µmol/L rotenone/antimycin A. The number of cells per well was determined using Seahorse XF imaging and cell counting software, and all OCR measurements were normalized to the number of cells in each well. Relative levels of fatty acid-driven basal and maximal mitochondrial respiration were calculated using Seahorse Wave software (Agilent).

### Detection of the activity of the TOPflash plasmid luciferase reporter gene in the WNT signalling pathway

293 T cells in the experimental group were cotransfected with the C6orf15 overexpression plasmid (GENECHEM, Shanghai), TOPflash luciferase reporter gene plasmid and Renilla plasmid for 48 h, and 293 T cells in the control group were cotransfected with the C6orf15 null plasmid and TOPflash luciferase reporter gene plasmid as well as the Renilla plasmid. The level of β-catenin-mediated TCF/LEF transcriptional activity in the WNT signalling pathway was detected by the Luciferase Reporter Gene Plasmid Kit (Beyotime Biotechnology, China) 48 h after transfection.

### Statistical analyses

The results of three independent experiments are expressed as the mean ± standard deviation. We performed all the statistical analyses using SPSS 17.0 statistical software (Chicago, IL, USA). Scientific plots were drawn using GraphPad Prism (GraphPad Software, USA). Student's t test was used to determine the relationship of C6orf15 expression with clinicopathological parameters and cell migration invasion. Pearson's correlation coefficient was used to explore the relationship between C6orf15 and β-catenin or ZEB1. Paired t tests were used to analyse C6orf15, β-catenin, ZEB1, Vimentin, N-cadherin, and E-cadherin protein expression. p < 0.05 was considered to indicate a statistically significant difference.

## Results

### C6orf15 is differentially expressed in CRC and is strongly associated with metastasis and poor prognosis

**Fig. 1:**
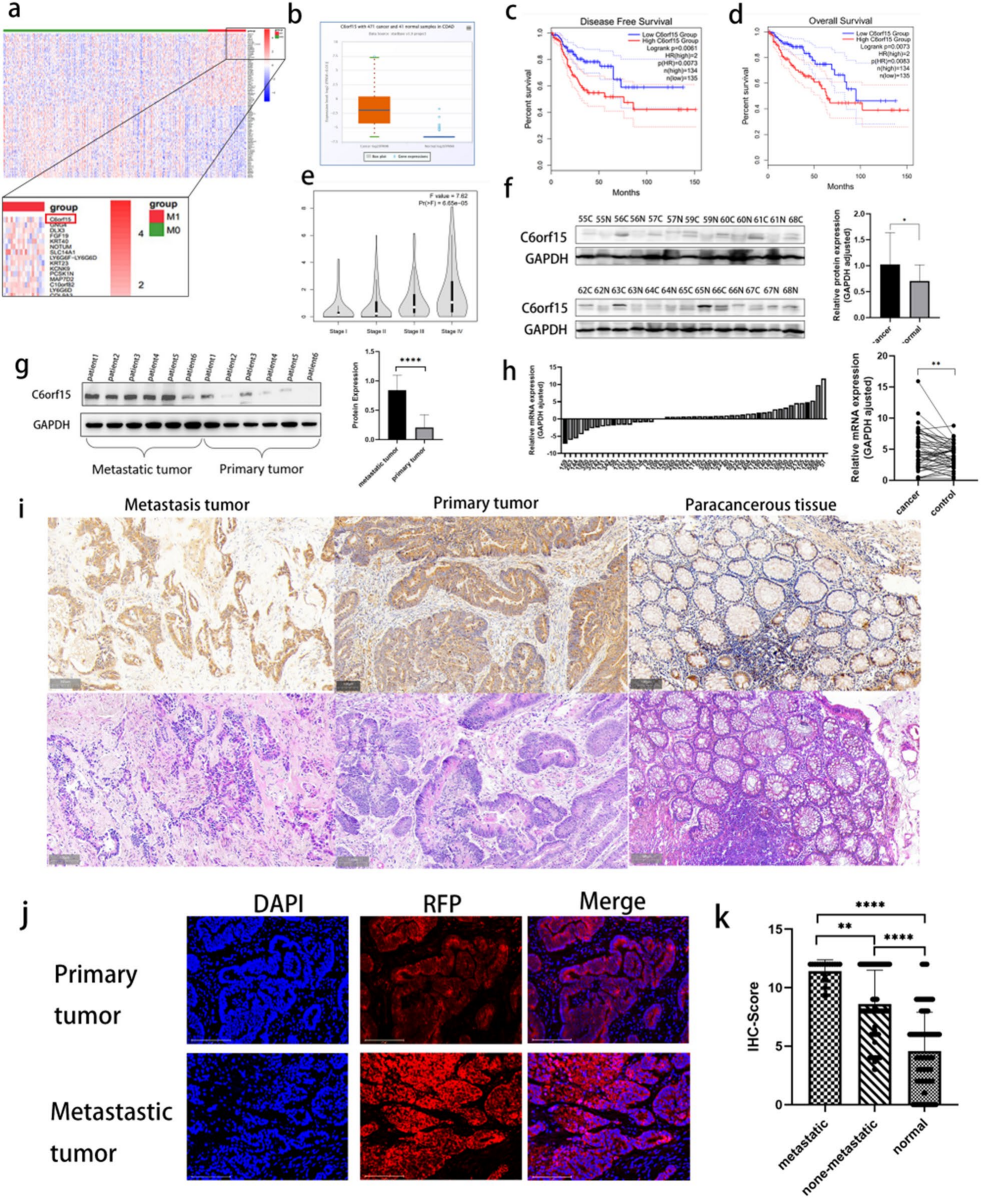
C6orf15 expression is associated with metastasis. **a** Data from the TCGA database; 312 tissues from patients with colon cancer without distant metastasis and 58 tissues from patients with colon cancer with distant metastasis were selected for differential expression analysis. **b** Prediction based on the Starbase database suggests that the expression of C6orf15 is higher in colon cancer than in normal intestinal tissue. **c** and **d** The GEPIA database was used to analyse the relationship between high and low C6orf15 expression and disease-free survival as well as overall survival. **e** The TCGA database was used to analyse C6orf15 expression differences in different stages of colon cancer. **f** Protein expression of C6orf15 in cancer and paracancerous tissues from 13 colon cancer patients (Western blots). **g** Expression of C6orf15 in liver metastatic lesions as well as intestinal primary lesions of 6 colorectal cancer patients who developed liver metastases (Western blots). **h** C6orf15 mRNA expression in 48 clinical tissue samples (qRT-PCR); the difference in mRNA expression between cancerous and adjacent tissues was assessed in these 48 patients. **i** Immunohistochemical staining of liver metastatic lesions, intestinal primary lesions and paracancerous tissues from colon cancer patients to visualize the expression of C6orf15 in different tissues. **j** Immunofluorescence was used to show C6orf15 expression in liver metastatic foci as well as primary foci of colon cancer tissues (shown in red fluorescence); nuclei were counterstained with DAPI (blue). **k** Scoring of 150 pairs of clinical tissue samples after immunohistochemical staining and statistical analysis. For all statistical results, *p < 0.05, **p < 0.01, ***p < 0.001, ****p < 0.0001, and ns (no statistical significance)

As shown in Fig. 1a and d, we conducted differential expression analysis using 312 samples from CRC patients without distant metastasis and 58 samples from CRC patients with distant metastasis selected from The Cancer Genome Atlas (TCGA, https://tcgadata.nci.nih.gov/tcga/). C6orf15 was the most significantly differentially expressed gene (|logFC|> 1, p value < 0.05). Similar conclusions have been reached in other related studies [10]. Concurrently, in our exploration of the TCGA database, we observed a significant correlation between C6orf15 expression and the CRC stage (|logFC|> 1, p value < 0.05). C6orf15 expression in stage IV tumours was notably greater than that in the preceding three stages (Fig. 1e), implying a potential association between elevated C6orf15 expression and CRC metastasis. GEPIA revealed that patients with high C6orf15 expression exhibited lower disease-free survival (DFS) and overall survival (OS) than patients with low C6orf15 expression (p < 0.05) (Fig. 1c). Moreover, C6orf15 expression may be higher in CRC tissues than adjacent tissues (Fig. 1b).

We conducted immunohistochemical staining and immunofluorescence analysis of 150 clinical tissue samples (collected from patients at the First Affiliated Hospital of China Medical University between 2005 and 2021). The results indicated greater expression of C6orf15 in liver metastases of CRC than in primary lesions, with elevated expression in cancerous tissues compared to adjacent noncancerous tissues (Fig. 1k). Additionally, western blot analysis of tissue samples from six patients with liver metastasis, both in metastatic and primary lesions, revealed significantly increased C6orf15 expression in metastatic lesions (Fig. 1g). Similar conclusions were drawn from the C6orf15 mRNA levels in 48 pairs of CRC tissues (Fig. 1h).Correlation analysis of the clinical data revealed that C6orf15 expression was closely associated with distant metastasis and lymph node metastasis in CRC patients (Additional file 1: Table S1).

The collective findings from the bioinformatics and tissue sample analyses suggest a close association among elevated C6orf15 expression and CRC metastasis, CRC stage and unfavourable prognosis. In summary, these results suggest that the upregulation of C6orf15 expression might be a necessary mechanism for CRC metastasis.

### C6orf15 promotes the development of liver metastasis in a mouse model of CRC

**Fig. 2:**
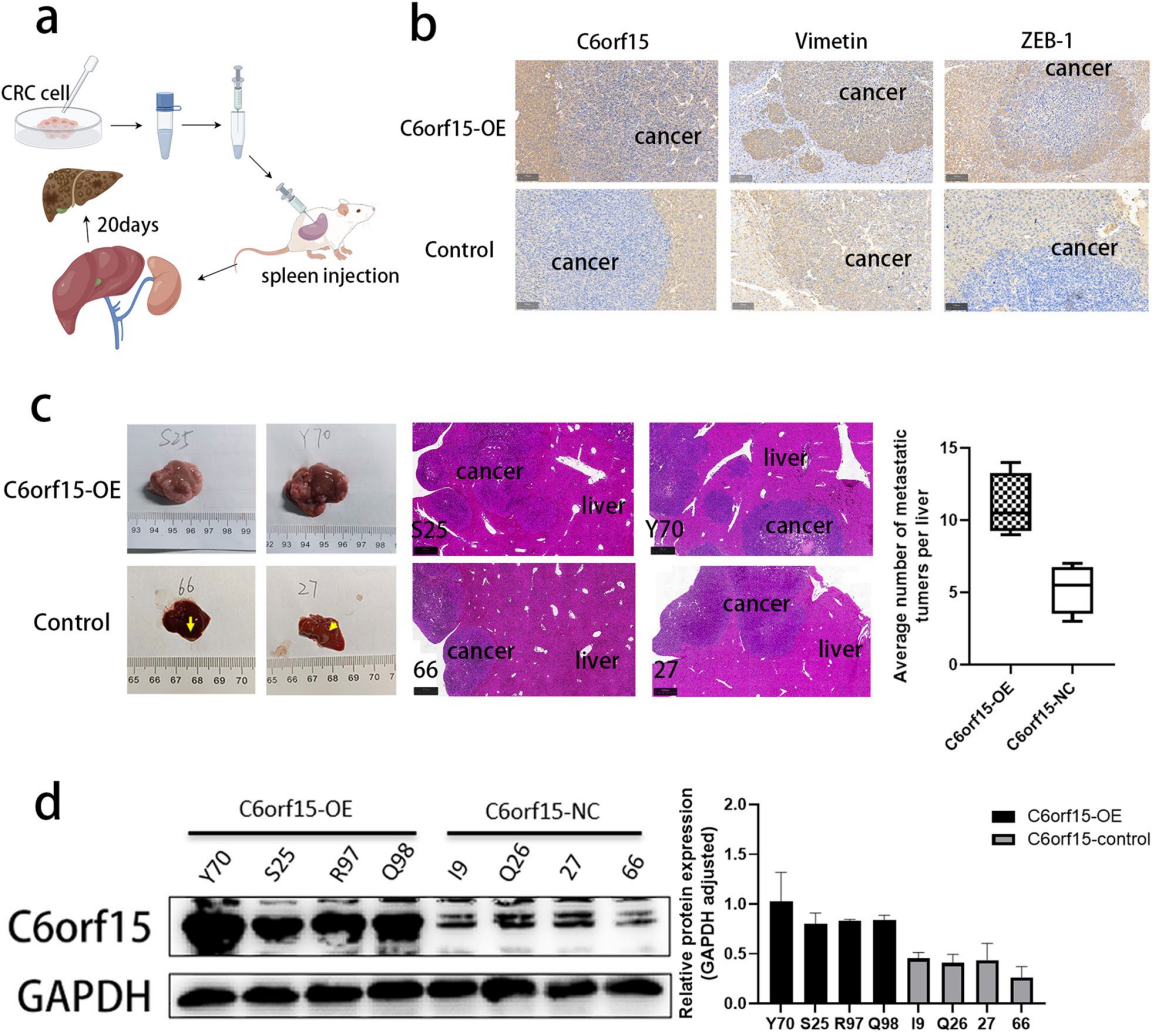
The upregulation of C6orf15 expression promotes the liver metastasis of CRC in mice. **a** Establishment of a mouse liver metastasis model via the splenic injection of C6orf15-overexpressing MC38 cells and control cells. **b** Immunohistochemical analysis of C6orf15, Vimentin and ZEB1 expression in the experimental and control groups. **c** Liver specimens from the two groups were collected and photographed to compare tumour formation (tumour size as well as number), and HE staining was used to visualize pathology. **d** Western blots show the expression of C6orf15 in tumour samples from both groups

To investigate the function of C6orf15 in vivo, we constructed a mouse colon cancer cell line, MC38, that stably overexpressed C6orf15 and assessed the effect of C6orf15 on the liver metastasis of CRC via a liver metastasis (splenic injection) model. As shown in Fig. 2c (Mouse Tumourigenesis), the overexpression group exhibited significantly greater numbers and larger sizes of liver metastases than the control group. Subsequently, we analysed the expression of C6orf15 and epithelial–mesenchymal transition (EMT) markers (Vimentin and ZEB1) in mouse liver metastases using immunohistochemistry (IHC) (Fig. 2b). The results indicated substantial increases in the expression levels of C6orf15, Vimentin and ZEB1 within metastatic lesions. These findings suggest that C6orf15 can promote liver metastasis in murine CRC.

### C6orf15 promotes invasion and proliferation and induces the epithelial–mesenchymal transition (EMT) in CRC

**Fig. 3:**
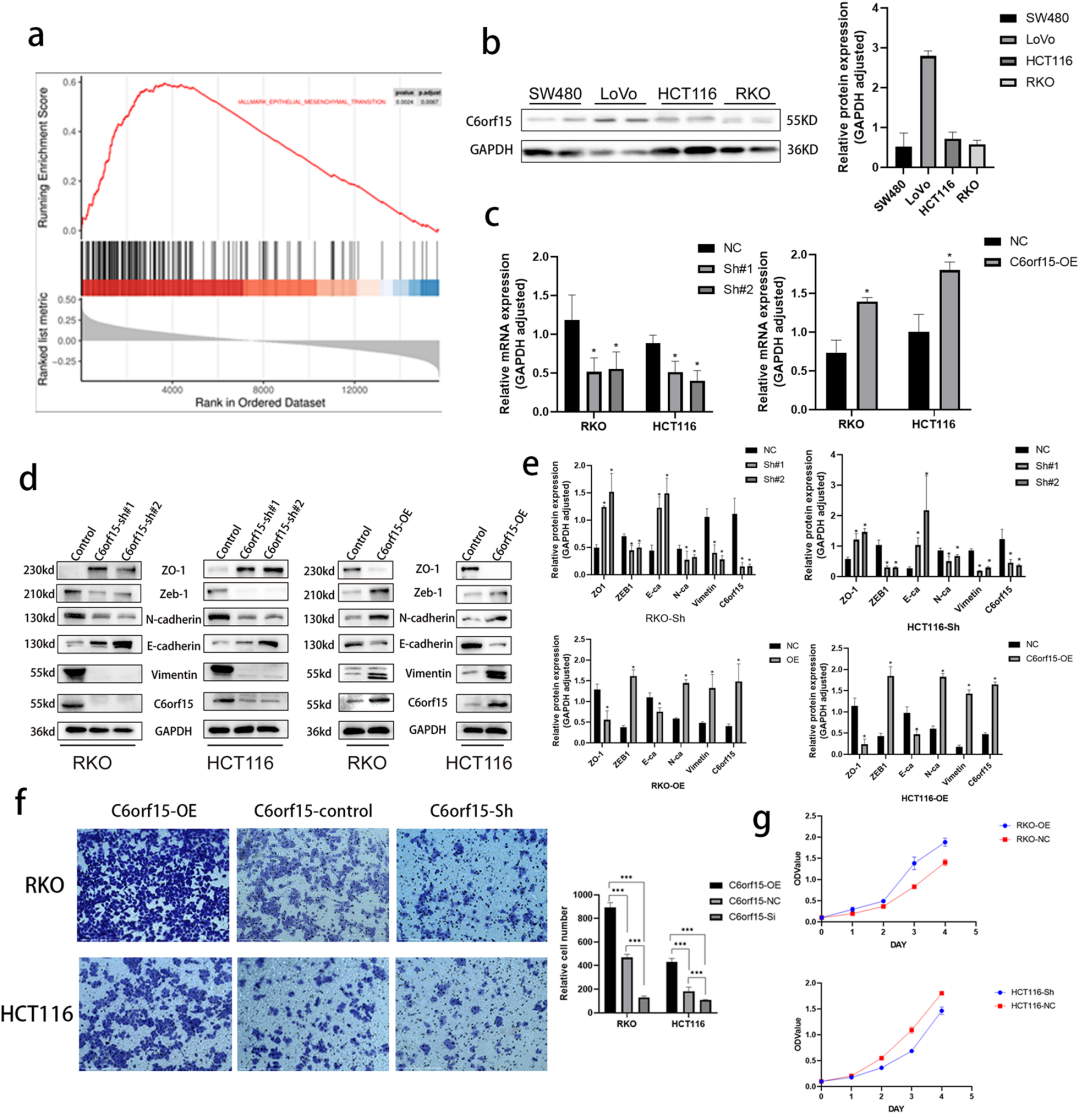
C6orf15 promotes invasion and proliferation and induces the epithelial–mesenchymal transition (EMT) in CRC. **a** Enrichment analysis of signalling pathways based on GSEA-Hallmark database. **b** Western blots show the expression of C6orf15 in different cell lines. **c** qRT-PCR was used to verify the gene expression efficiency after the transfection of lentiviruses to silence or overexpress C6orf15. **d** and **e** Western blots show the expression changes of EMT-related markers after upregulating and downregulating C6orf15 expression. **f** Transwell assay results show that C6orf15 overexpression enhanced CRC migration ability and C6orf15 downregulation reduced CRC migration potential. **g** MTT assays were used to determine the proliferation ability of RKO cells and HCT116 cells transfected with lentiviruses to silence or overexpress C6orf15. For all statistical results, *p < 0.05, **p < 0.01, ***p < 0.001, ****p < 0.0001, and ns (no statistical significance)

We identified a significant correlation between C6orf15 and the epithelial–mesenchymal transition (EMT) through the Hallmark database. Next, we successfully constructed stable HCT116 and RKO cell lines in which C6orf15 was overexpressed or knocked down via lentiviral transfection (Fig. 3c). Subsequent Transwell assays confirmed that the overexpression of C6orf15 enhanced the invasive capabilities of the cell lines, whereas C6orf15 silencing inhibited the invasion of HCT116 and RKO cells (Fig. 3f). Western blot analysis of key EMT molecules revealed that silencing C6orf15 increased E-cadherin and ZO-1 expression while reducing ZEB-1, Vimentin, and N-cadherin expression. Conversely, C6orf15 overexpression inhibited E-cadherin and ZO-1 expression and promoted ZEB-1, Vimentin, and N-cadherin expression (Fig. 3d and e). Zeb1 binds to the regulatory gene motif in the E-box and has been rigorously proven to be one of the major regulatory proteins of the EMT [11]. Zeb1 expression is frequently upregulated in CRC and regulates the EMT directly through binding to the E-box on the E-cadherin promoter region or regulated the EMT through the recruitment of the corepressor C-terminal binding protein [12]. Both mechanisms result in the downregulation of E-cadherin expression. In addition to activating EMT transcription factors at the genomic level, the EMT is ultimately executed through two independent dynamic events: (1) the downregulation of the epithelial adhesion protein E-cadherin, which leads to unstable cell adhesion, and (2) an increase in mesenchymal protein products (such as N-cadherin and vimentin), which drive cell motility and invasion [13]. MTT assay results demonstrated a significant increase in the proliferative capacity of stable CRC-overexpressing cells compared to that of control cells. Conversely, CRC cell proliferation decreased after C6orf15 silencing (Fig. 3g). Consequently, we concluded that C6orf15 can induce the EMT in CRC, thereby enhancing the invasive and proliferative abilities of CRC cells.

### C6orf15 activates the WNT/β-catenin signalling pathway, promoting the nuclear translocation of β-catenin

**Fig. 4:**
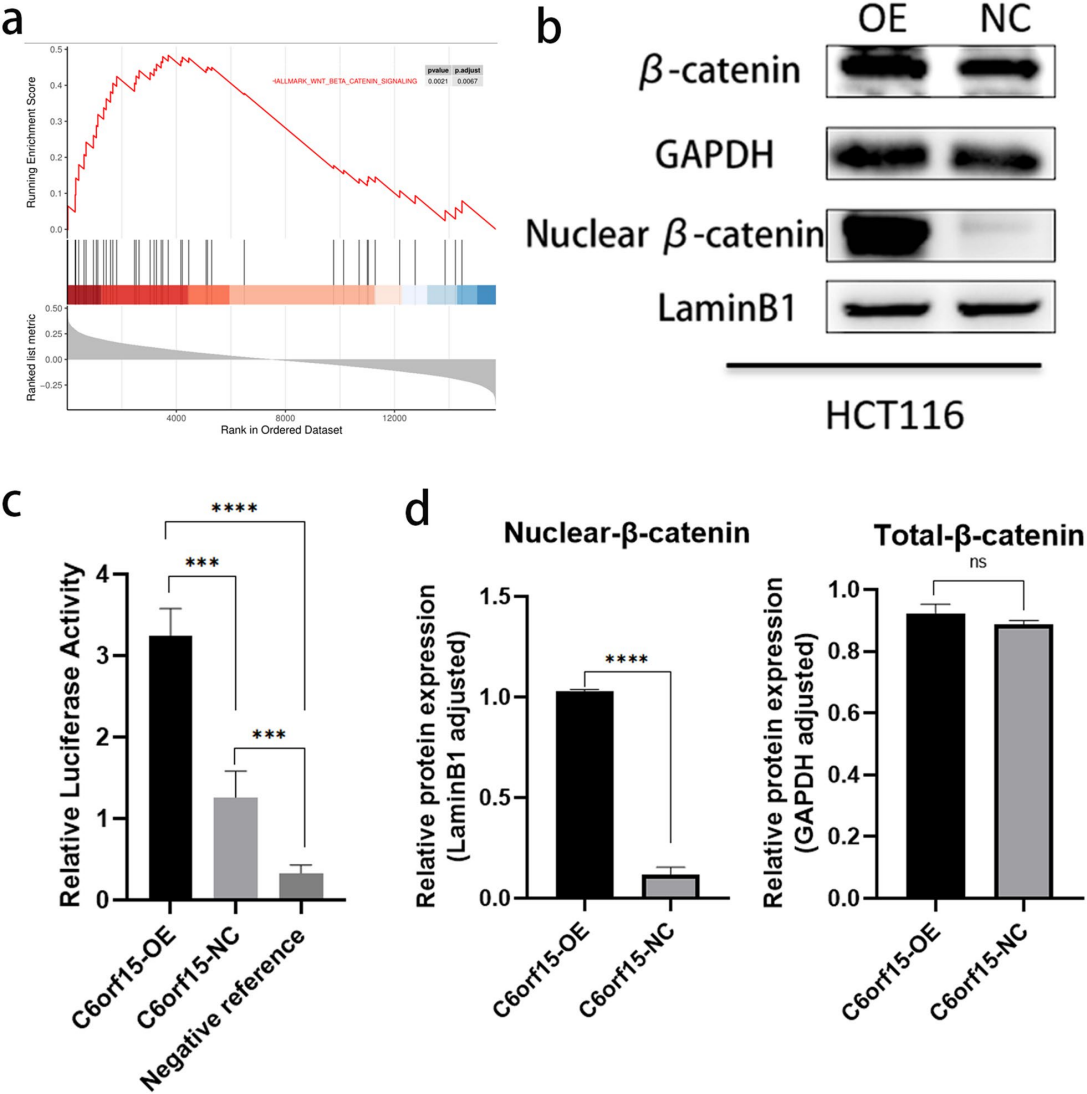
C6orf15 activates the WNT/β-catenin signalling pathway, promoting the nuclear translocation of β-catenin. **a** Signalling pathway enrichment analysis based on GSEA-Hallmark data showed that C6orf15 was positively correlated with the WNT signalling pathway. **b** and **d** Western blot results indicate that the upregulation of C6orf15 expression promotes β-catenin nuclear translocation. **c** TOPflash-Luciferase reporter gene assay results show that TCF/LEF1 transcriptional activity significantly increased after C6orf15 overexpression. For all statistical results, *p < 0.05, **p < 0.01, ***p < 0.001, ****p < 0.0001, and ns (no statistical significance)

To investigate the molecular mechanisms by which C6orf15 influences the EMT in CRC cells, we conducted an enrichment analysis of signalling pathways using the Hallmark database (Fig. 4a). The WNT signalling pathway has emerged as one of the most highly relevant pathways. Existing research suggests that the activation of the WNT/β-catenin signalling pathway induces EMT progression in CRC by altering cell‒cell adhesion and promoting invasiveness. The key to WNT signalling pathway activation is the nuclear accumulation of β-catenin. In our study, western blot results from the stable C6orf15-overexpressing HCT116 cell line revealed significant nuclear translocation of β-catenin, although the total protein level of β-catenin did not show a notable increase.

Subsequently, we cotransfected 293 T cells with C6orf15-overexpression plasmids, the TOPflash luciferase reporter gene plasmid, and the Renilla plasmid. After 48 h of incubation, we measured TCF/LEF-mediated transcriptional activity in the WNT signalling pathway. The results showed a tenfold increase in transcriptional activity in the overexpression group compared to that in the control group. Therefore, C6orf15 can activate the WNT/β-catenin signalling pathway and promote the nuclear translocation of β-catenin, resulting in a significant increase in TCF/LEF transcription, thereby facilitating the initiation of the EMT in CRC.

### C6orf15 can affect long-chain fatty acid metabolism in CRC through WNT/β-catenin signalling

**Fig. 5:**
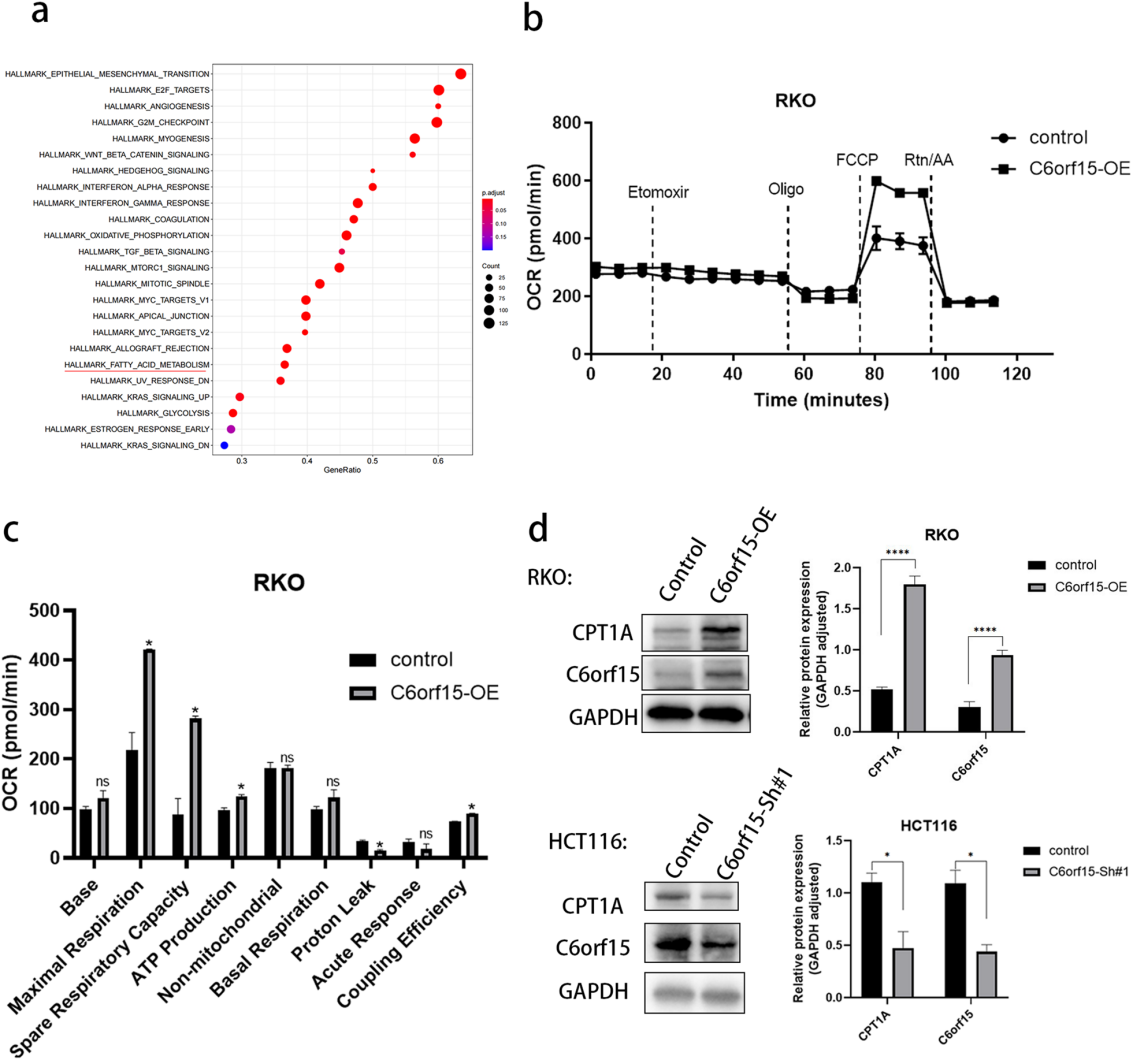
C6orf15 can affect long-chain fatty acid metabolism in CRC through WNT/β-catenin signalling. **a** The GSEA-Hallmark database indicates a strong correlation between C6orf15 and fatty acid oxidation. **b** Control and C6orf15-OE RKO cells were cultured in substrate-limited medium, followed by FAO testing using the Seahorse XF96 Cell Output Analyser as described in the Materials and Methods section. Etomoxir, oligo, FCCP, and antimycotic A (anti-A) were added sequentially as indicated by the dotted line. OCR measurements were normalized to the total number of cells. **c** Fatty acid-driven mitochondrial respiration was calculated from OCR measurements and used to reflect basal and maximal levels of fatty acid oxidation. The results are shown as the mean ± SD (*p < 0.05). **d** Western blots show the protein expression of CPT1A in C6orf15-overexpressing CRC and C6orf15-silenced CRC. For all statistical results, *p < 0.05, **p < 0.01, ***p < 0.001, ****p < 0.0001, and ns (no statistical significance)

**Fig. 6:**
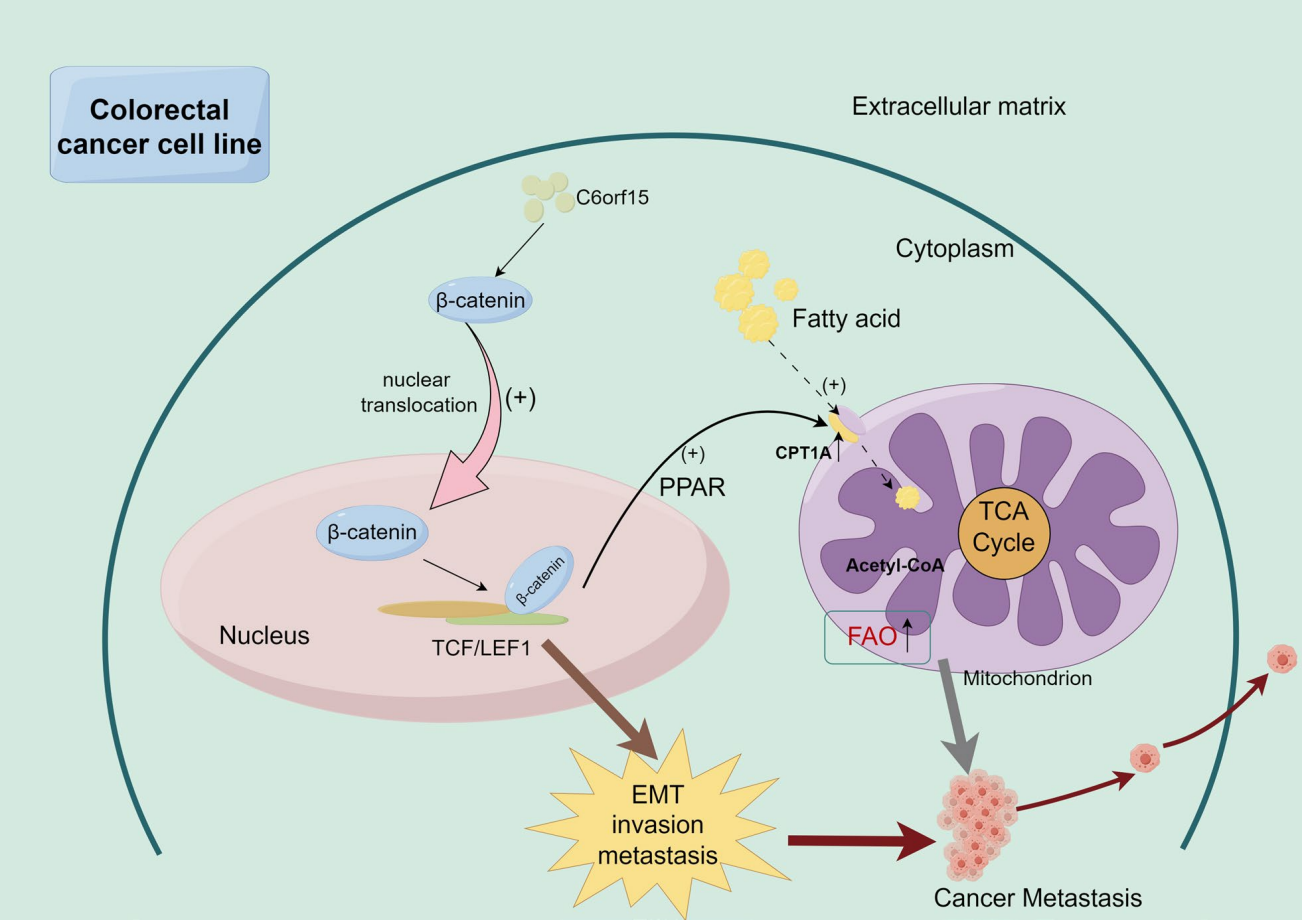
The proposed mechanism by which C6orf15 activates the WNT/β-catenin signalling pathway to promote the EMT and fatty acid metabolism in colorectal cancer

Research on metabolism has gained considerable attention, particularly in the context of CRC, over the last few years. Several studies have indicated a close association between fatty acid metabolism and the occurrence of liver metastasis in colorectal cancer [14, 15]. The impact of fatty acid metabolism on tumour proliferation and metastasis is multifaceted and involves aspects such as energy storage, membrane proliferation, and engagement as signalling molecules in tumour activities [16]. CPT1A is a key regulator that connects the adipocyte-mediated regulation of cellular metabolism to WNT signalling in colon cancer cells [17]. Previous research has demonstrated that the activation of the WNT signalling pathway can enhance the transcription-dependent expression of PPARδ, thereby promoting CPT1A expression. In this study, based on an analysis of the GSEA and Hallmark databases, there was a significant correlation between C6orf15 and FAO (Fig. 5a). Seahorse XF96 extracellular flux analysis was performed using long-chain fatty acids as metabolic substrates to analyse the metabolic profiles of control cells and CRC cells overexpressing the C6orf15 gene (Fig. 5b and c). The findings revealed a significant increase in the oxygen consumption rate (OCR) associated with maximal respiration, spare respiratory capacity, ATP production, proton leakage, and coupling efficiency in C6orf15-overexpressing RKO cells, confirming the role of C6orf15 in controlling mitochondrial FAO. Moreover, western blotting revealed a corresponding increase in the expression of carnitine palmitoyl transferase 1A (CPT1A) in C6orf15-overexpressing RKO cells. CPT1A is a key enzyme in FAO. The upregulation of CPT1A expression promotes FAO. In previous experiments, the upregulation of C6orf15 expression was shown to activate WNT signalling, promoting the nuclear aggregation of β-catenin. Consequently, we hypothesized that C6orf15 may influence fatty acid metabolism in CRC through the WNT/β-catenin signalling pathway, thereby impacting CRC proliferation or metastasis (Fig. 6).

## Discussion

Despite multifaceted advances in the treatment of CRC, it remains one of the most aggressive malignancies and is characterized by rapid tumour recurrence and early metastasis [18]. Liver metastases are the leading cause of death in colorectal cancer patients. Despite advancements in modern medical technology, including surgical procedures and neoadjuvant therapy, which can improve patient prognosis, overall outcomes remain unstable, and the mortality rate due to liver metastasis remains high. Therefore, there is an urgent need to explore new treatment targets. Previous relevant studies C6orf15 promotes the occurrence and development of colon cancer by promoting the ECM receptor interaction pathway, Hedgehog signalling pathway and WNT signaling pathway^10^. However, they did not elucidate how C6orf15 impacts CRC metastasis, whereas our study provides new insights into the mechanistic aspects.

The function of C6orf15 has not been clearly elucidated, and previous studies have noted that it is expressed in the skin and tonsils [19], which may be associated with susceptibility to follicular lymphoma. Moreover, its absence may be associated with desquamation [20]. C6orf15 is predicted to be involved in extracellular matrix composition and collagen V- and fibronectin-binding activities. These functions might be relevant to the EMT biological process in CRC, although the role of this gene in solid tumours has not been reported.

Ectopic WNT activation is common in human cancers, especially in CRC. WNT signalling plays a key role in developmental processes, such as affecting stem cell proliferation and differentiation [21]. The aberrant activation of typical WNT signalling leads to the nuclear translocation of β-catenin in dedifferentiated mesenchymal-like tumour cells undergoing active EMT associated with downregulated E-cadherin expression. The activation of typical WNT signalling pathways, including that of transcription factors such as TWiST1, ZEB1 and MMP9, has been reported to initiate the aberrant activation of the EMT [22]. In contrast, DKK1, a WNT signalling protein inhibitor, suppresses the malignant biological behaviour of CRC cells by inhibiting the EMT programme [23]. The activation of WNT signalling in CRC occurs through the inactivation of APC or mutation of β-catenin. A key step in both processes is the nuclear accumulation of β-catenin, which promotes the EMT.

Previous results indicate that β-catenin activation induces EMT progression by altering intercellular junctions, leading to the invasiveness of CRC [24]. Lymphoid enhancer binding factor 1 (LEF1) is a member of the high-mobility transcription factor T-cell factor (TCF)/LEF1 family. It serves as a downstream mediator of the WNT/β-catenin signalling pathway, but it can also independently regulate gene transcription. LEF1 is essential for stem cell maintenance and organ development, particularly through the activation of the transcription of signature EMT effectors involved in the EMT, including N-cadherin, Vimentin, and snail [25]. The nuclear aggregation of β-catenin is a crucial step in initiating the transcription of LEF1-regulated genes. In our study, we found that the upregulation of C6orf15 expression promoted β-catenin nuclear translocation and nuclear aggregation and significantly enhanced TCF/LEF1 transcriptional activity. Moreover, western blot results showed that the expression of the transcription factor ZEB1 increased/decreased in C6orf15-overexpressing/knockdown CRC cells, and the expression of E-cadherin, N-cadherin and Vimentin (the Hallmark effector proteins of the EMT) showed corresponding changes. Hence, we postulate that the upregulation of C6orf15 expression activates the WNT signalling pathway, initiates β-catenin nuclear translocation, triggers downstream TCF/LEF1 transcription, increases ZEB1 transcription, and stimulates EMT programme activation, leading to the EMT in CRC cells. The specific mechanism by which C6orf15 induces β-catenin nuclear translocation requires further exploration, but based on previous studies, increased intracellular calcium in breast cancer cells leads to β-catenin nuclear translocation [26], which may also be a potential mechanism by which C6orf15 promotes β-catenin nuclear translocation. Additionally, the ubiquitination degradation pathway of GSK-3β-phosphorylated β-catenin might be inhibited, possibly being another key factor leading to β-catenin nuclear translocation [27].

Several existing epidemiological studies have provided strong evidence that obesity and colon cancer development are inextricably linked [28–30]. However, the molecular mechanisms by which fatty acids support tumour growth and progression remain largely unknown. CPT1A was proposed to be a key regulator linking fatty cell-mediated cell metabolism regulation to WNT signal transduction in colon cancer cells. The activation of WNT signalling can cause the nuclear translocation of β-catenin, promote downstream TCF/LEF1 transcription, and further activate the PPARδ-dependent transcription of CPT1A, resulting in the enhancement of fatty acid metabolic pathways [31]. In our study, C6orf15 activated the WNT signalling pathway and promoted the nuclear translocation of β-catenin to induce the EMT in colon cancer, and coincidentally, we found an increase in the FAO rate and an increase in the expression of CPT1A after C6orf15 overexpression. According to previous studies, there is a consensus that fatty acid metabolism is strongly associated with the EMT and metastasis in colon cancer [14, 32]. Therefore, it is reasonable to speculate that the upregulation of C6orf15 expression activates the WNT signalling pathway, which promotes β-catenin entry into the nucleus, enhances downstream TCF/LEF1 transcription, increases CPT1A and ZEB1 expression, and ultimately promotes liver metastasis in CRC.

## Conclusion

Our study identified C6orf15 as a marker of liver metastasis in CRC. C6orf15 can activate the WNT/β-catenin signalling pathway to promote the EMT and fatty acid metabolism in CRC, but the specific molecular mechanism needs to be further investigated. Nevertheless, identifying C6orf15 as a notable marker for CRC liver metastasis provides a solid foundation for its use as a molecular target for CRC treatment.

### Supplementary Information

**Additional file 1: Table S1.** Association of C6orf15 Expression Levels with Clinical Data. **Table S2.** List of antibodies used in the study. **Table S3.** Primers used for RT-PCR in this study.

## Data Availability

Any reasonable requests for access to available data underlying the results reported in this article will be considered. Such proposals should be submitted to the corresponding author.
