# Supplementary material for: C6orf15 promotes liver metastasis via WNT/β-catenin signalling in colorectal cancer
Source: Cancer Cell Int. 2024 Apr 23;24:146. doi: 10.1186/s12935-024-03324-2 (PMC11040941; doi:10.1186/s12935-024-03324-2)
Supplement: Supplementary file 1 — Additional file 1: Table S1. Association of C6orf15 Expression Levels with Clinical Data. Table S2. List of antibodies used in the study. Table S3. Primers used for RT-PCR in this study. [file 12935_2024_3324_MOESM1_ESM.docx]

**Table S1:** Association of C6orf15 Expression Levels with Clinical Data

The results showed that C6orf15 expression correlated with the presence of CEA and TNM stage of colon cancer. (*p<0.05)

| parameters | No. of Patients | High expression | Low expression | P value |
| --- | --- | --- | --- | --- |
| Ages(years) |  |  |  |  |
| <60  ≥60 | 108  42 | 64  22 | 44  20 | 0.4444 |
| Gender |  |  |  |  |
| Male  Female | 73  77 | 43  43 | 30  34 | 0.7049 |
| Tumor Size(maximal diameter) |  |  |  |  |
| ≥5cm  ≤5cm | 54  96 | 36  50 | 18  46 | 0.0830 |
| Differentiation |  |  |  |  |
| Well, Moderate  Poor | 102  48 | 53  33 | 49  15 | 0.0525 |
| CEA |  |  |  |  |
| (+)  (-) | 94  56 | 62  24 | 32  32 | 0.0057** |
| Metastasis |  |  |  |  |
| M0  M1 | 116  34 | 60  26 | 56  8 | 0.0109* |
| Lymph node Metastasis |  |  |  |  |
| N0  N+ | 64  86 | 29  57 | 35  29 | 0.0125* |

**Table S2:** List of antibodies used in the study.

| **Antibodies** | **Cat. Number** | **Manufacturers** |
| --- | --- | --- |
| C6orf15 | 24953-1-AP | Protein-tech North America |
| ZEB1 | T57181S | Abmart Shanghai |
| E-cadherin | [TA0131](http://www.ab-mart.com.cn/page.aspx?node=%2077%20&id=%2022176) | Abmart Shanghai |
| N-cadherin | [TU391037](http://www.ab-mart.com.cn/page.aspx?node=%2077%20&id=%20110296) | Abmart Shanghai |
| ZO-1 | [TA5145](http://www.ab-mart.com.cn/page.aspx?node=%2077%20&id=%2021809) | Abmart Shanghai |
| Vimentin | 10366-1-AP | Protein-tech North America |
| GAPDH | [M20006](http://www.ab-mart.com.cn/page.aspx?node=%2059%20&id=%20984) | Abmart Shanghai |
| β-catenin | 51067-2-AP | Protein-tech North America |
| CPT1A | 15184-1-AP | Protein-tech North America |
| LaminB1 | 12987-1-AP | Protein-tech North America |

**Table S3:** Primers used for RT-PCR in this study.

| **Target** | **Forward 5’ → 3’** | **Reverse 5’→ 3’** |
| --- | --- | --- |
| C6orf15 | TGCTCCTGGTCTGTCTTCATCTCC | GGCTGCGGATGTTCAGAGTTAGAG |
| GAPDH | CAGGAGGCATTGCTGATGAT | GAAGGCTGGGGCTCATTT |

Thermal cycling conditions were: 95ºC for 3 minutes followed by 45 cycles of 95ºC for 12 seconds and 62ºC for 45 seconds.
